# Pooled safety results across phase 3 randomized trials of intravenous golimumab in rheumatoid arthritis, psoriatic arthritis, and ankylosing spondylitis

**DOI:** 10.1186/s13075-022-02753-6

**Published:** 2022-03-21

**Authors:** M. Elaine Husni, Atul Deodhar, Sergio Schwartzman, Soumya D. Chakravarty, Elizabeth C. Hsia, Jocelyn H. Leu, Yiying Zhou, Kim H. Lo, Arthur Kavanaugh

**Affiliations:** 1 Department of Rheumatic and Immunologic Diseases, Clinical Outcomes Research, Arthritis and Musculoskeletal Center, Cleveland Clinic, Desk A50, 9500 Euclid Ave., Cleveland, OH 44195 USA; 2 Oregon Health & Science University, Portland, OR USA; 3 Weill Cornell Medical College, New York, NY USA; 4 Janssen Scientific Affairs, LLC, Horsham, PA USA; 5 Drexel University College of Medicine, Philadelphia, PA USA; 6 Janssen Research & Development, LLC, Spring House, PA USA; 7 University of Pennsylvania, Philadelphia, PA USA; 8 University of California San Diego, La Jolla, CA USA

**Keywords:** Intravenous golimumab, TNF inhibitor, Rheumatoid arthritis, Psoriatic arthritis, Ankylosing spondylitis, Safety, Adverse event

## Abstract

**Background:**

Intravenous (IV) golimumab, a TNFi, is approved for treating rheumatoid arthritis (RA), psoriatic arthritis (PsA), and ankylosing spondylitis (AS). We analyzed pooled safety results from three phase 3 IV golimumab trials in these rheumatologic diseases and hypothesized that the safety profile of IV golimumab would be similar to that established for other TNFi, including subcutaneous golimumab.

**Methods:**

Data from three double-blind, randomized trials of IV golimumab in patients with RA, PsA, and AS, each with a placebo-controlled period and an extension of active treatment, were included. Golimumab 2 mg/kg was administered at weeks 0 and 4, then every 8 weeks through week 100 (RA) or week 52 (PsA, AS). Concomitant low-dose, oral corticosteroids were permitted. Concomitant methotrexate was required in the RA trial and permitted in the PsA and AS trials; placebo patients crossed over to golimumab at weeks 24 (RA, PsA) and 16 (AS), respectively. Adverse events (AEs), including infections, serious infections, malignancies, and major adverse cardiovascular events (MACE), were assessed through week 112 (RA) or week 60 (PsA, AS).

**Results:**

In total, 539 patients were randomized to placebo, and 740 patients were randomized to golimumab; 1248 patients received ≥ 1 golimumab administration. Among the placebo and golimumab patients, respectively, during the placebo-controlled periods, 40.6% and 50.3% had an AE, 2.4% and 3.8% had a serious AE, and 0.4% and 0.8% had a serious infection. Among all golimumab-treated patients, the numbers of events/100 patient-years (95% CI) were as follows: AEs, 175.2 (169.0, 181.6); serious AEs, 12.7 (11.0, 14.5); serious infections, 3.4 (2.5, 4.4); active tuberculosis, 0.4 (0.1, 0.8); opportunistic infection, 0.2 (0.1, 0.6); malignancies, 0.4 (0.2, 0.9), and MACE, 0.5 (0.2, 1.0). There were no cases of lymphoma. Three (0.6%) placebo-treated patients and 6 (0.5%) golimumab-treated patients died during the studies. Concomitant methotrexate was associated with increased occurrence of elevated alanine transaminase levels and lower incidence of antibodies to golimumab. During the placebo-controlled periods, serious infections in the placebo and golimumab groups were more common in patients receiving concomitant low-dose oral corticosteroids vs. those not receiving corticosteroids.

**Conclusions:**

IV golimumab demonstrated a safety profile that was broadly consistent across these rheumatologic indications and with other TNFi, including subcutaneous golimumab. Concomitant methotrexate or corticosteroids were associated with an increase in specific AEs.

**Trial registrations:**

ClinicalTrials.gov, NCT00973479. Registered on September 9, 2009. ClinicalTrials.gov, NCT02181673. Registered on July 4, 2014. ClinicalTrials.gov, NCT02186873. Registered on July 10, 2014.

## Introduction

Golimumab is a human monoclonal antibody that binds and inhibits the pro-inflammatory cytokine tumor necrosis factor alpha (TNFα). Intravenous (IV) golimumab is approved for the treatment of rheumatoid arthritis (RA) (in combination with methotrexate [MTX]), psoriatic arthritis (PsA), and ankylosing spondylitis (AS) [1]. Efficacy and safety of IV golimumab have been assessed in three double-blind, randomized, placebo-controlled, phase 3 trials of patients with RA (GO-FURTHER) [2–4], PsA (GO-VIBRANT) [5, 6], and AS (GO-ALIVE) [7, 8]. Patients with immune-mediated diseases such as RA, PsA, and AS are likely to require ongoing treatment. Thus, the long-term safety of treatments for these conditions is of particular interest for both health care providers and patients. Possible associations with the risk of serious infections, opportunistic infections, tuberculosis (TB), demyelinating disorders, and lymphoma have been reported for other TNF inhibitors (TNFi) [9–14].

Intravenous golimumab has demonstrated significantly greater improvements, compared with placebo, in signs and symptoms in patients with RA [2], PsA [5, 15], and AS [7]. In addition, IV golimumab treatment has slowed radiographic progression compared with placebo in patients with RA and PsA [3, 15]. Trial extensions of active treatment with golimumab demonstrated that improvements were maintained over time [4, 6, 8].

**Fig. 1:**
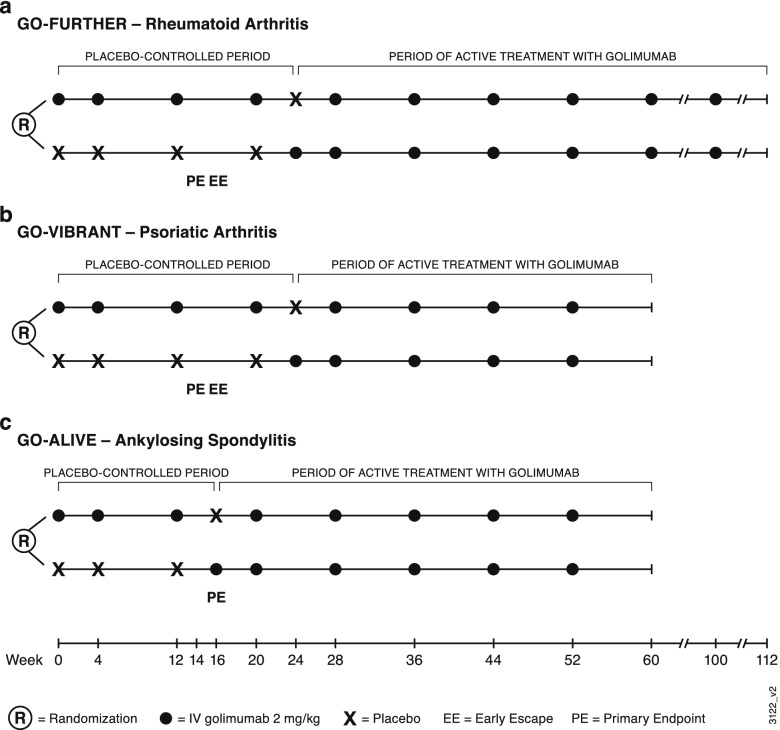
Study designs of three phase 3 trials of golimumab in patients with **a** rheumatoid arthritis (GO-FURTHER), **b** psoriatic arthritis (GO-VIBRANT), and **c** ankylosing spondylitis (GO-ALIVE)

Here, we report pooled safety results from the three double-blind, randomized, placebo-controlled, phase 3 trials of IV golimumab in patients with RA [2–4], PsA [5, 6], and AS [7, 8], each with a placebo-controlled period and an extension of active treatment with golimumab (Fig. 1). Safety results are presented for all treated patients, as well as for patient subgroups defined by baseline use of MTX and low-dose oral corticosteroids.

## Methods

### Patient populations and study designs

These analyses utilized data from three double-blind, randomized, placebo-controlled, phase 3 trials of IV golimumab: GO-FURTHER (NCT00973479), GO-VIBRANT (NCT02181673), and GO-ALIVE (NCT02186873) (Fig. 1). In GO-FURTHER, eligible adults with active RA (≥ 6/66 swollen joints and ≥ 6/68 tender joints) despite MTX therapy (≥ 3 months) were randomized (2:1) to receive IV golimumab 2 mg/kg at weeks 0 and 4 and every 8 weeks thereafter through week 100 or placebo infusions at weeks 0 and 4 and every 8 weeks with crossover to golimumab at week 24 [2]. Patients in the placebo group who had < 10% improvement in swollen and tender joint counts entered early escape (double-blind) at week 16 and received IV golimumab 2 mg/kg. Patients were required to continue their stable doses of concomitant MTX (15–25 mg/week) and were permitted to continue oral low-dose corticosteroid therapy (≤ 10 mg/day of prednisone or equivalent) throughout the trial.

In GO-VIBRANT, adult patients with active PsA (≥ 5/66 swollen joints, ≥ 5/68 tender joints, and C-reactive protein ≥ 0.6 mg/dL) were eligible [5]. Patients were randomized (1:1) to receive IV golimumab 2 mg/kg at weeks 0 and 4 and every 8 weeks thereafter through week 52 or placebo infusions at weeks 0 and 4 and every 8 weeks with crossover to golimumab at week 24. Concomitant MTX (≤ 25 mg/week) was permitted for those patients who had been receiving MTX for ≥ 3 months at a stable dose for ≥ 4 weeks. At week 16, patients in both groups who had < 5% improvement in swollen and tender joint counts could enter early escape and were allowed to have one of the following treatment changes at the investigators’ discretion: an increase in corticosteroid dose (total dose ≤ 10 mg/day prednisone or equivalent), MTX dose (total dose ≤ 25 mg/week), or non-steroidal anti-inflammatory drugs (NSAIDs) dose; or initiation of NSAIDs, corticosteroids (≤ 10 mg/day prednisone or equivalent), MTX (≤ 25 mg/week), sulfasalazine (≤ 3 g/day), hydroxychloroquine (≤ 400 mg/day), or leflunomide (≤ 20 mg/day).

The GO-ALIVE trial included adults with active AS (Bath Ankylosing Spondylitis Disease Activity Index [BASDAI] ≥ 4 and a visual analog scale [0–10 cm] score for total back pain of ≥ 4) [7]. Patients were randomized (1:1) to receive IV golimumab 2 mg/kg at weeks 0 and 4 and every 8 weeks thereafter through week 52 or placebo infusions at weeks 0 and 4 with crossover to golimumab at week 16. Patients could have received prior therapy with one TNFi other than golimumab (limited to 20% of enrollment) ≥ 3 months before the first study agent administration. Stable doses of concomitant MTX (≤ 25 mg/week), sulfasalazine, hydroxychloroquine, NSAIDs, other analgesics, and low-dose oral corticosteroids (dose equivalent to ≤ 10 mg prednisone/day) were permitted.

In all three trials, infusions were administered over approximately 30 min. Placebo-treated patients received only saline solution that did not contain excipients found in the golimumab formulation. Infusion reactions were defined as adverse events (AEs) that occurred within 1 h following infusion of the study agent. Patients were permitted prophylactic drugs (excluding corticosteroids) prior to infusion; pre-treatment was at the discretion of the investigator and typically included acetaminophen, antihistamines, and/or NSAIDs.

The trials were conducted in accordance with the principles of the Declaration of Helsinki and Good Clinical Practices. Protocols were approved by an Institutional Review Board or ethics committees for each site, and all patients provided written informed consent.

### Assessments

Safety was monitored throughout the trials, with final safety assessments at week 112 in GO-FURTHER and week 60 in GO-VIBRANT and GO-ALIVE or at specified times after early discontinuation (~ 12 weeks in the RA trial and 8 weeks in the PsA and AS trials). Serum samples were collected for laboratory analyses and for the detection of antibodies to golimumab. A highly sensitive, drug-tolerant, enzyme immunoassay was used to detect antibodies to golimumab [16]. Immunogenicity analyses included all golimumab-treated patients who had a baseline sample and at least one post-baseline golimumab infusion.

### Statistical methods

Adverse events are summarized by treatment group within each study and pooled across the studies for both the placebo-controlled period and open-label follow-up. Due to the differences in the lengths of follow-up between the three IV golimumab trials, time-adjusted incidences of AEs (events per 100 patient-years [100 PY]) with 95% confidence intervals are also provided across indications by treatment groups. Adverse events of interest included serious infections, malignancies, major adverse cardiovascular events (MACE, defined as cardiovascular death, non-fatal myocardial infarction, and non-fatal stroke), and infusion reactions.

## Results

### Patients

In the combined RA, PsA, and AS trials, 1279 patients were randomized and received ≥ 1 administration of placebo (*n* = 539) or IV golimumab (*n* = 740). A total of 1248 patients received ≥ 1 golimumab administration, including those who were randomized to placebo and initiated IV golimumab at pre-specified time points for crossover or early escape (Fig. 1). Baseline demographic and disease characteristics have been reported in each study [2, 5, 7] with selected characteristics shown in Table 1 for reference. Patients with RA had a higher mean age than patients with PsA or AS, and there were fewer men than women in the RA trial and fewer women than men in the AS trial. MTX use varied by trial; at baseline, 75% of patients were receiving MTX and 45% were receiving oral corticosteroids. All patients received MTX in the RA trial by design, while 70% in the PsA trial were receiving MTX at baseline. A minority (18%) of patients in the AS trial were receiving MTX at baseline. Corticosteroid use at baseline was also higher in the RA trial (65.0%) compared with the PsA (27.7%) and AS (26.4%) trials (Table 1).

**Table 1:** Baseline demographics and disease characteristics for patients enrolled in studies of IV golimumab in RA, PsA, and AS*

	RA trial		PsA trial		AS trial	
	Placebo	Golimumab	Placebo	Golimumab	Placebo	Golimumab
Patients, *N*	197	395	239	241	103	105
Demographics and disease characteristics						
Mean age, years (range)	51.4 (19, 78)	51.9 (18, 83)	46.7 (18, 79)	45.7 (19, 69)	39.2 (20, 67)	38.4 (19, 64)
Male, *n* (%)	40 (20.3)	69 (17.5)	121 (50.6)	128 (53.1)	77 (74.8)	86 (81.9)
BMI, kg/m^2^	27.0 (5.7)	26.8 (5.5)	28.9 (6.2)	28.9 (6.4)	26.8 (6.4)	27.2 (5.9)
Disease duration, years	7.0 (7.2)	6.9 (7.0)	5.3 (5.9)	6.2 (6.0)	5.5 (5.9)	5.6 (6.6)
Swollen Joint count (0–66)	14.8 (8.5)	15.0 (8.2)	14.1 (8.2)	14.0 (8.4)	–	–
Tender joint count (0–68)	25.9 (14.1)	26.4 (13.9)	26.1 (14.4)	25.1 (13.8)	–	–
CRP, mg/dL	2.2 (1.9)	2.8 (2.9)	2.0 (2.0)	1.9 (2.5)	1.9 (1.7)	2.0 (1.8)
BASDAI, *N*	–	–	53**	56**	103	105
Score	–	–	6.4 (1.9)	6.5 (1.8)	7.1 (1.2)	7.0 (1.2)
Methotrexate, *n* (%)	197 (100)	395 (100)	173 (72.4)	163 (67.6)	21 (20.4)	16 (15.2)
Dose, mg/week	16.6 (2.8)	16.8 (2.9)	14.9 (4.8)	14.8 (4.7)	13.7 (5.0)	16.7 (4.9)
Oral corticosteroids, *n* (%)	134 (68.0)	251 (63.5)	67 (28.0)	66 (27.4)	23 (22.3)	32 (30.5)
Dose***, mg/day	7.0 (2.5)	7.0 (2.5)	7.6 (2.5)	7.4 (2.6)	6.1 (2.5)	7.8 (2.7)
NSAIDs, *n* (%)	156 (79.2)	323 (81.8)	167 (69.9)	173 (71.8)	90 (87.4)	94 (89.5)

*AS* ankylosing spondylitis, *BASDAI* Bath Ankylosing Spondylitis Disease Activity Index, *BMI* body mass index, *CRP* C-reactive protein, *IV* intravenous, *NSAID* non-steroidal anti-inflammatory drug, *PsA* psoriatic arthritis, *RA* rheumatoid arthritis, *SD* standard deviation

*Data presented as mean (SD) unless otherwise noted

**Among patients with investigator-assessed spondylitis in addition to peripheral arthritis as their primary presentation of PsA

***Prednisone or equivalent

Detailed patient disposition through the end of the studies has been previously reported [2–8]. In total, 1104 (86%) patients who received ≥ 1 golimumab administration (including 468 placebo ➔ golimumab patients) completed study treatment through week 52/100. In these pooled analyses, the mean duration of follow-up ranged from 16 to 23 weeks for placebo and 47 to 96 weeks for golimumab. The number of IV administrations per patient (mean ± SD) was 3.9 ± 0.8 for placebo and 9.0 ± 3.6 for golimumab. For golimumab-treated patients, the total PY of exposure used to determine the incidence/100 PY was 1077 in the RA trial, 417 in the PsA trial, and 203 in the AS trial for a pooled total PY of 1697.

### Adverse events during the placebo-controlled periods of the IV golimumab trials

Across the trials, during the placebo-controlled periods, 40.6% of placebo-treated patients and 50.3% of IV golimumab-treated patients experienced ≥ 1 AE, and 2.4% and 3.8% respectively had ≥ 1 serious AE (SAE) (Table 2). Details for each study have been previously reported [2, 5, 7]. Across the three studies, a greater proportion of golimumab-treated patients discontinued treatment because of an AE (2.3%) compared with placebo (0.7%; Table 2). Infections were the most common type of AE. Through the placebo-controlled periods, infections and serious infections, respectively, occurred in 17.3% and 0.4% of placebo patients and in 23.8% and 0.8% of golimumab-treated patients (Table 2). Adverse events reported in ≥ 3% of patients in any treatment group included upper respiratory tract infection, nasopharyngitis, and increases in serum aminotransferases (Table 2). Most SAEs were singular events, with no clear relationship to the underlying conditions or study treatment across the three trials. Three deaths occurred during the placebo-controlled periods of the trials, all among patients receiving placebo and none in the golimumab groups: in the RA trial, one death due to stroke; in the PsA trial, one each due to acute cardiac failure subsequent to pneumonia and cardiorespiratory insufficiency due to metastasis (esophageal neoplasm) [2, 5].

**Table 2:** Adverse events during the placebo-controlled periods of studies of IV golimumab in patients with RA, PsA, and AS*

	RA trial, weeks 0–24		PsA trial, weeks 0–24		AS trial, weeks 0–16		Pooled	
	Placebo	Golimumab	Placebo	Golimumab	Placebo	Golimumab	Placebo	Golimumab
Patients, *N*	197	395	239	240	103	105	539	740
Mean follow-up, weeks	20.9	23.6	23.2	23.9	16.0	16.1	21.0	22.6
Patients who discontinued due to an AE	1 (0.5)	12 (3.0)	3 (1.3)	5 (2.1)	0	0	4 (0.7)	17 (2.3)
Patients with ≥ 1 AE	98 (49.7)	227 (57.5)	97 (40.6)	111 (46.3)	24 (23.3)	34 (32.4)	219 (40.6)	372 (50.3)
Patients with ≥ 1 infection	48 (24.4)	119 (30.1)	37 (15.5)	45 (18.8)	8 (7.8)	12 (11.4)	93 (17.3)	176 (23.8)
Patients with ≥ 1 SAE	5 (2.5)	19 (4.8)	8 (3.3)	7 (2.9)	0	2 (1.9)	13 (2.4)	28 (3.8)
Patients with ≥ 1 serious infection	0	4 (1.0)	2 (0.8)	1 (0.4)	0	1 (1.0)	2 (0.4)	6 (0.8)
Patients with malignancy	0	1 (0.3)	2 (0.8)	0	0	0	2 (0.4)	1 (0.1)
Deaths	1 (0.5)	0	2 (0.8)	0	0	0	3 (0.6)	0
Patients with ≥ 1 infusion reaction	1 (0.5)	14 (3.5)	0	4 (1.7)	0	3 (2.9)	1 (0.2)	21 (2.8)
Common AEs**								
Upper respiratory tract infection	15 (7.6)	29 (7.3)	3 (1.3)	7 (2.9)	1 (1.0)	3 (2.9)	19 (3.5)	39 (5.3)
Increased ALT	7 (3.6)	11 (2.8)	5 (2.1)	19 (7.9)	0	3 (2.9)	12 (2.2)	33 (4.5)
Headache	5 (2.5)	20 (5.1)	5 (2.1)	5 (2.1)	1 (1.0)	4 (3.8)	11 (2.0)	29 (3.9)
Nasopharyngitis	5 (2.5)	10 (2.5)	13 (5.4)	8 (3.3)	1 (1.0)	6 (5.7)	19 (3.5)	24 (3.2)
Increased AST	3 (1.5)	7 (1.8)	5 (2.1)	13 (5.4)	0	0	8 (1.5)	20 (2.7)
Hypertension	4 (2.0)	14 (3.5)	4 (1.7)	4 (1.7)	0	1 (1.0)	8 (1.5)	19 (2.6)
Bronchitis	2 (1.0)	12 (3.0)	4 (1.7)	1 (0.4)	0	0	6 (1.1)	13 (1.8)
Urinary tract infection	6 (3.0)	12 (3.0)	0	0	1 (1.0)	0	7 (1.3)	12 (1.6)
Worsening rheumatoid arthritis	12 (6.1)	9 (2.3)	0	0	0	0	12 (2.2)	9 (1.2)
Neutropenia	0	0	5 (2.1)	9 (3.8)	0	0	5 (0.9)	9 (1.2)

*AE* adverse event, *AS* ankylosing spondylitis, *ALT* alanine aminotransferase, *AST* aspartate aminotransferase, *IV* intravenous, *PsA* psoriatic arthritis, *RA* rheumatoid arthritis, *SAE* serious adverse event

*Data presented as *n* (%) unless otherwise noted

**AEs occurring in ≥ 3% of patients in any treatment group

### Adverse events in IV golimumab-treated patients over the course of treatment

Through completion of the studies, the numbers/100 PY of AEs, SAEs, and discontinuations due to AEs were numerically greater in the RA trial, followed by the PsA and then AS trials (Table 3). Altogether, discontinuation due to an AE during treatment with IV golimumab occurred at a rate of 5% over the course of all trials.

**Table 3:** Adverse events through study completion in patients with RA, PsA, and AS who received ≥ 1 administration of IV golimumab*

	RA trial	PsA trial	AS trial	Pooled golimumab
Patients, *N*	584	460	204	1248
Mean follow-up, weeks (SD)	95.9 (25.7)	47.2 (13.0)	51.8 (9.0)	70.7 (30.7)
Patient years of follow-up	1077	417	203	1697
Patients who discontinued due to an AE	41 (7.0)	17 (3.7)	4 (2.0)	62 (5.0)
AEs				
Patients with ≥ 1 AE	462 (79.1)	234 (50.9)	113 (55.4)	809 (64.8)
Events/100 patient-years (95% CI)	195.4 (187.2, 204.0)	143.5 (132.3, 155.5)	133.0 (117.6, 149.8)	175.2 (169.0, 181.6)
Infections				
Patients with ≥ 1 infection	287 (49.1)	105 (22.8)	67 (32.8)	459 (36.8)
Events/100 patient-years (95% CI)	59.9 (55.4, 64.7)	34.0 (28.7, 40.1)	49.8 (40.5, 60.5)	52.3 (48.9, 55.9)
SAEs				
Patients with ≥ 1 SAE	106 (18.2)	24 (5.2)	7 (3.4)	137 (11.0)
Events/100 patient-years (95% CI)	16.1 (13.8, 18.7)	8.2 (5.6, 11.4)	3.9 (1.7, 7.8)	12.7 (11.0, 14.5)
Serious infections				
Patients with ≥ 1 serious infection	36 (6.2)	10 (2.2)	3 (1.5)	49 (3.9)
Events/100 patient-years (95% CI)	4.0 (2.9, 5.4)	2.6 (1.3, 4.7)	1.5 (0.3, 4.3)	3.4 (2.5, 4.4)
MACE				
Patients with ≥ 1 MACE	9 (1.5)	0	0	9 (0.7)
Events/100 patient-years (95% CI)	0.8 (0.4, 1.6)	0 (0.0, 0.7)	0 (0.0, 1.5)	0.5 (0.2, 1.0)
Malignancies				
Patients	5 (0.9)	2 (0.4)	0	7 (0.6)
Events/100 patient-years (95% CI)	0.5 (0.2, 1.1)	0.5 (0.1, 1.7)	0 (0.0, 1.5)	0.4 (0.2, 0.9)
Deaths				
Patients	5 (0.9)	1 (0.2)	0	6 (0.5)
Events/100 patient-years (95% CI)	0.5 (0.2, 1.1)	0.2 (0.0, 1.3)	0 (0.0, 1.5)	0.4 (0.1, 0.8)
Opportunistic infections				
Patients with ≥ 1 opportunistic infection	4 (0.7)	0	0	4 (0.3)
Events/100 patient-years (95% CI)	0.4 (0.1, 1.0)	0 (0.0, 0.7)	0 (0.0, 1.5)	0.2 (0.1, 0.6)
Active TB				
Patients	3 (0.5)	2 (0.4)	1 (0.5)	6 (0.5)
Events/100 patient-years (95% CI)	0.3 (0.1, 0.8)	0.5 (0.1, 1.7)	0.5 (0.0, 2.7)	0.4 (0.1, 0.8)
Infusion reaction				
Patients with ≥ 1 infusion reaction	27 (4.6)	4 (0.9)	3 (1.5)	34 (2.7)
Events/100 patient-years (95% CI)	3.7 (2.7, 5.1)	1.4 (0.5, 3.1)	2.0 (0.5, 5.0)	3.0 (2.2, 3.9)

*AE* adverse event, *AS* ankylosing spondylitis, *CI* confidence interval, *IV* intravenous, *MACE* major adverse cardiovascular event, *PsA* psoriatic arthritis, *RA* rheumatoid arthritis, *SAE* serious adverse event, *SD* standard deviation, *TB* tuberculosis

*Data presented as *n* (%) unless otherwise noted

Six deaths occurred in golimumab-treated patients, and all occurred after the placebo-controlled periods: pneumonia with subsequent presumed myocardial infarction, acute abdominal syndrome (abscess fluid removed during laparotomy was positive for TB), septic shock secondary to a pyogenic lung abscess, dehydration due to *Clostridium difficile*, and one of unknown cause in the RA trial [2–4] and one due to acute hepatitis of mixed etiology in the PsA trial [6]. No deaths occurred in the AS trial [7, 8].

### Adverse events of interest

Malignancies occurred in 2 (0.4%) patients receiving placebo (non-small cell lung cancer and esophageal neoplasm in the PsA trial; Table 2). In addition, one case of non-treatment-emergent lung adenocarcinoma occurred in a patient randomized to placebo in the RA trial. Among IV golimumab-treated patients, eight malignancies occurred in seven (0.6%) patients: basal cell carcinoma, breast cancer, cervical carcinoma in situ, and chronic lymphocytic leukemia, and one patient with both Bowen’s disease and basal cell carcinoma (all in the RA trial) and gastric cancer and colon cancer (one patient each) in the PsA trial. The incidence of malignancies/100 PY (95% CI) across the three studies was 0.4 (0.2, 0.9) (Table 3). There were no cases of lymphoma.

In total, 49 (3.9%) of all golimumab-treated patients had a serious infection; the incidence/100 PY (95% CI) was 3.4 (2.5, 4.4) across the three trials (4.0 [2.9, 5.4] in the RA trial; 2.6 [1.3, 4.7] in the PsA trial, and 1.5 [0.3, 4.3] in the AS trial; Table 3). Serious infections included pneumonia (*n* = 11), urinary tract infection (*n* = 5), sepsis (*n* = 4), appendicitis (*n* = 2), empyema (*n* = 2), and erysipelas (*n* = 2); other serious infections were singular events and included infected dermal cyst, acute pyelonephritis, periodontitis, and acute hepatitis of mixed etiology [4, 6, 8]. Among the golimumab-treated patients, six cases of active TB occurred (RA trial, *n* = 3; PsA trial, *n* = 2; AS trial, *n* = 1) (Table 3)), all in patients who screened negative for TB at baseline and lived in countries endemic for TB (Argentina, Lithuania, Malaysia, Mexico, and Ukraine) [4, 6, 8]. No opportunistic infections occurred in the PsA or AS trials. Four opportunistic infections occurred among golimumab-treated patients in the RA trial: cryptococcal pneumonia (*n* = 1) and localized vertebral candidiasis (*n* = 1) (both classified as serious and led to discontinuation of golimumab) [4], and two golimumab-treated patients were diagnosed with non-serious esophageal candidiasis infection.

One demyelinating event (non-infectious encephalomyelitis) occurred in a patient receiving golimumab in the PsA trial [5]. Two events of uveitis occurred in patients who were receiving placebo (both in the AS trial). One golimumab-treated patient had de novo uveitis (RA trial); the patient had a concurrent herpes simplex infection of the eye. Two patients reported pregnancy during the trial, both randomized to golimumab: one patient (RA trial) discontinued study treatment with no additional information provided, and the other patient (PsA trial) discontinued study treatment and had an elective termination. There were no cases of drug-induced lupus or aplastic anemia.

Two patients experienced heart failure (both in the PsA trial): one patient in the placebo group developed acute heart failure that had a fatal outcome, and one patient in the golimumab group had a non-serious AE of chronic heart failure (hypertension and ischemic heart disease ongoing at baseline; the patient discontinued study treatment). Two patients experienced deep vein thrombosis: one golimumab-treated patient in the RA trial and one placebo-treated patient in the PsA trial. The incidence (95% CI) of MACE among all golimumab-treated patients was 0.5/100 PY (0.2, 1.0), all occurring in the RA trial (Table 3): two deaths (one due to unknown cause and the aforementioned presumed myocardial infarction secondary to pneumonia), three non-fatal myocardial infarctions, and four non-fatal strokes. The incidence (95% CI) of MACE among patients receiving placebo was 1.4/100 PY (0.3, 4.0): two deaths (the aforementioned stroke [RA trial] and acute cardiac failure subsequent to pneumonia [PsA trial]) and one non-fatal stroke (PsA trial) [2, 5].

During the placebo-controlled periods, infusion reactions were seen in 2.8% of all IV golimumab-treated patients (Table 2); the incidence remained low over the course of the trials (3.0/100 PY; Table 3). No infusion reaction was considered serious or severe. One patient in the PsA trial receiving golimumab discontinued study agent due to chest tightness associated with infusion.

### Adverse events in patients with and without concomitant methotrexate or oral corticosteroids

Across the three trials, 965 (placebo, *n* = 391; golimumab, *n* = 574) were receiving concomitant MTX at baseline (Table 4). Concomitant MTX treatment was mandatory in the RA trial and optional in the PsA and AS trials. During the placebo-controlled periods of the PsA and AS trials, infections, serious infections, and SAEs occurred in comparable proportions of patients receiving vs. not receiving concomitant MTX (Table 4). Pooling data across the three trials, infections appeared to be more common in patients with vs. without MTX use at baseline during the placebo-controlled periods and through trial completion. However, interpretation of these findings is limited by the requirement for all patients in the RA trial to use concomitant MTX. Through the end of the PsA trial, concomitant MTX treatment was associated with elevations in serum hepatic alanine aminotransferase (ALT) from ≥ 3 to < 5 times the upper limit of normal (ULN); the occurrence of ALT elevations ≥ 5× ULN was similar between patients with and without MTX use (Table 4). No clear relationship was seen between MTX use and aspartate aminotransferase elevation (data not shown).

**Table 4:** Adverse events, including clinical laboratory abnormalities, summarized by methotrexate use at baseline in patients with RA, PsA, and AS*

Occurrence during placebo-controlled periods, *n* (%)														
	RA trial		PsA trial				AS trial				Pooled			
	PBO	Golimumab	PBO		Golimumab		PBO		Golimumab		PBO		Golimumab	
Methotrexate use at baseline	+	+	+	−	+	−	+	−	+	−	+	−	+	−
Treated patients, *N*	197	395	173	66	163	77	21	82	16	89	391	148	574	166
Mean follow-up, weeks	20.9	23.6	23.2	23.1	23.8	24.0	15.9	16.0	16.1	16.1	21.6	19.2	23.5	19.7
≥ 1 infection	48 (24.4)	119 (30.1)	26 (15.0)	11 (16.7)	26 (16.0)	19 (24.7)	2 (9.5)	6 (7.3)	2 (12.5)	10 (11.2)	76 (19.4)	17 (11.5)	147 (25.6)	29 (17.5)
≥ 1 SAE	5 (2.5)	19 (4.8)	4 (2.3)	4 (6.1)	5 (3.1)	2 (2.6)	0	0	0	2 (2.2)	9 (2.3)	4 (2.7)	24 (4.2)	4 (2.4)
≥ 1 serious infection	0	4 (1.0)	1 (0.6)	1 (1.5)	1 (0.6)	0	0	0	0	1 (1.1)	1 (0.3)	1 (0.7)	5 (0.9)	1 (0.6)
Baseline ALT ≤ ULN, *n*	182	357	144	60	135	74	18	76	15	83	344	136	507	157
ALT ≥ 3 to < 5× ULN	2 (1.1)	4 (1.1)	1 (0.7)	0	2 (1.5)	1 (1.4)	0	0	0	1 (1.2)	3 (0.9)	0	6 (1.2)	2 (1.3)
ALT ≥ 5× ULN	0	3 (0.8)	0	1 (1.7)	0	1 (1.4)	0	1 (1.3)	0	0	0	2 (1.5)	3 (0.6)	1 (0.6)
Baseline ALT > ULN, *n*	14	34	25	4	27	3	3	4	1	6	42	8	62	9
ALT ≥ 3 to < 5× ULN	3 (21.4)	4 (11.8)	0	0	3 (11.1)	1 (33.3)	0	0	0	0	3 (7.1)	0	7 (11.3)	1 (11.1)
ALT ≥ 5× ULN	0	0	0	0	3 (11.1)	0	0	0	0	0	0	0	3 (4.8)	0
Events/100 patient-years through study completion**														
	RA trial		PsA trial				AS trial				Pooled			
	Golimumab		Golimumab				Golimumab				Golimumab			
Methotrexate use at baseline	+		+		−		+		−		+		−	
Patients, *N*	584		323		137		36		168		943		305	
Total PY of follow-up	1077		290		127		35		168		1402		295	
Serious infections	4.0 (2.9, 5.4)		3.8 (1.9, 6.8)		0 (0.0, 2.4)		0 (0.0, 8.5)		1.8 (0.4, 5.2)		3.9 (2.9, 5.0)		1.0 (0.2, 3.0)	
ALT ≥ 3 to < 5× ULN	3.5 (2.5, 4.8)		21.0 (16.1, 27.0)		7.9 (3.8, 14.5)		2.8 (0.1, 15.8)		2.4 (0.7, 6.1)		7.1 (5.8, 8.7)		4.8 (2.6, 8.0)	
ALT ≥ 5× ULN	2.0 (1.3 ,3.1)		3.1 (1.4, 5.9)		3.2 (0.9, 8.1)		0 (0.0, 8.5)		1.2 (0.1, 4.3)		2.2 (1.5, 3.1)		2.0 (0.8, 4.4)	
Patients positive for antibodies to golimumab***, *n*/*N* (%)	129/552 (23.4)		60/316 (19.0)		39/134 (29.1)		4/36 (11.1)		37/167 (22.2)		193/904 (21.3)		76/301 (25.2)	

“+” indicates with methotrexate; “−” indicates without methotrexate

*AE* adverse event, *ALT* alanine aminotransferase, *AS* ankylosing spondylitis, *CI* confidence interval, *PBO* placebo, *PsA* psoriatic arthritis, *PY* patient-years, *RA* rheumatoid arthritis, *SAE* serious adverse event, *ULN* upper limit of normal

*Data presented as *n* (%), *n*/*N* (%), or events /100 PY (95% CI), unless otherwise noted

**The three trials ranged from 60 to 112 weeks in duration (Fig. 1). Time-adjusted incidence of AEs (events/100 PY) are shown

***Antibodies to golimumab were assessed using a drug-tolerant enzyme immunoassay at 52 weeks for all three trials

A total of 573 (placebo, *n* = 224; golimumab, *n* = 349) patients were receiving concomitant low-dose oral corticosteroids at baseline (Table 5). Concomitant treatment with oral corticosteroids was associated with an increased rate of SAEs during the placebo-controlled period of the RA trial (Table 5). However, the trend did not continue through the extended course of golimumab treatment in the RA trial, and SAEs were not more common in patients receiving corticosteroid treatment in the PsA and AS trials. Among the pooled golimumab-treated patients, the incidence of serious infections per 100 PY (95% CI) through the end of the studies was similar between patients who received concomitant oral corticosteroids (3.4 [2.2, 4.8]) and those who did not (3.4 [2.2, 4.9]).

**Table 5:** Adverse events summarized by corticosteroid use at baseline in patients with RA, PsA, and AS*

Occurrence during placebo-controlled periods, *n* (%)																
	RA trial				PsA trial				AS trial				Pooled			
	PBO		Golimumab		PBO		Golimumab		PBO		Golimumab		PBO		Golimumab	
Corticosteroid use at baseline	+	-	+	−	+	−	+	−	+	−	+	−	+	−	+	−
Treated patients, *N*	134	63	251	144	67	172	66	174	23	80	32	73	224	315	349	391
Mean follow-up, weeks	21.1	20.4	23.5	23.9	22.7	23.3	23.4	24.0	15.9	16.0	16.0	16.1	21.0	20.9	22.8	22.5
≥ 1 infection	34 (25.4)	14 (22.2)	79 (31.5)	40 (27.8)	9 (13.4)	28 (16.3)	13 (19.7)	32 (18.4)	0	8 (10.0)	2 (6.3)	10 (13.7)	43 (19.2)	50 (15.9)	94 (26.9)	82 (21.0)
≥ 1 SAE	5 (3.7)	0	16 (6.4)	3 (2.1)	3 (4.5)	5 (2.9)	1 (1.5)	6 (3.4)	0	0	0	2 (2.7)	8 (3.6)	5 (1.6)	17 (4.9)	11 (2.8)
≥ 1 serious infection	0	0	3 (1.2)	1 (0.7)	2 (3.0)	0	1 (1.5)	0	0	0	0	1 (1.4)	2 (0.9)	0	4 (1.1)	2 (0.5)
Events/100 patient-years through study completion**																
	RA trial				PsA trial				AS trial				Pooled			
	Golimumab				Golimumab				Golimumab				Golimumab			
Corticosteroid use at baseline	+		−		+		−		+		−		+		−	
Patients, *N*	381		203		126		334		55		149		562		686	
Total PY of follow-up	697		379		112		306		57		146		865		832	
SAE	16.4 (13.5, 19.6)		15.6 (11.8, 20.1)		4.5 (1.5, 10.5)		9.5 (6.4, 13.6)		0 (0.0, 5.3)		5.5 (2.4, 10.8)		13.8 (11.4, 16.5)		11.5 (9.4, 14.1)	
Serious infections	3.6 (2.3, 5.3)		4.7 (2.8, 7.5)		3.6 (1.0, 9.2)		2.3 (0.9, 4.7)		0.0 (0.0, 5.3)		2.1 (0.4, 6.0)		3.4 (2.2, 4.8)		3.4 (2.2, 4.9)	

“+” indicates with corticosteroids; “−” indicates without corticosteroids

*AE* adverse event, *AS* ankylosing spondylitis, *CI* confidence interval, *PBO* placebo, *PsA* psoriatic arthritis, *PY* patient-years, *RA* rheumatoid arthritis, *SAE* serious adverse event

*Data presented as *n* (%) or events/100 PY (95% CI), unless otherwise noted

**The three trials ranged from 60 to 112 weeks in duration (Fig. 1). Time-adjusted incidence of AEs (events/100 PY) is shown

### Immunogenicity

In total, 1205 patients received ≥ 1 administration of IV golimumab and had appropriate samples for evaluating immunogenicity. Through week 52 of all three trials, 269 (22%) patients were positive for antibodies to golimumab (RA trial, 23%; PsA trial, 22%; AS trial, 20%), including 71 (6%) who were positive for neutralizing antibodies. Infusion reactions occurred in 20 (2.1%) patients who tested negative for antibodies to golimumab and in 11 (4.1%) who tested positive for antibodies to golimumab; the proportions of infusions with an infusion reaction were 0.4% and 0.8%, respectively. In the PsA and AS trials, in which not all patients received MTX, the occurrence of antibodies to golimumab was lower in patients who were receiving MTX at baseline compared with patients who were not (Table 4); a similar trend was observed for neutralizing antibodies (data not shown).

## Discussion

Patients with RA [17], PsA [18, 19], and AS [20, 21] typically require life-long treatment; therefore, long-term safety is an important consideration for both health care providers and patients when selecting treatment. In these pooled analyses, we combined the safety results of the TNFi IV golimumab 2 mg/kg from three phase 3 studies, GO-FURTHER (RA), GO-VIBRANT (PsA), and GO-ALIVE (AS). The proportion of patients who completed the safety follow-up (through week 112 in the RA trial and week 60 in the PsA and AS trials) was relatively high (86%), resulting in a total of 1697 PY of exposure. During these trials, IV golimumab was generally well-tolerated, and AEs were consistent with the known safety profile of other TNFi, including subcutaneous golimumab [9, 11, 13, 14, 22].

During the placebo-controlled periods, SAEs were uncommon in both the placebo group (2.4%) and the combined golimumab group (3.8%). Infections were the most common type of AE in the pooled results, which was consistent with the results reported individually from each of these studies. The incidence of serious infections per 100 PY was highest in the RA trial, followed by the PsA and AS trials; the pooled incidence of 3.4 serious infections per 100 PY in all golimumab-treated patients was comparable to that reported for other TNFi agents [23–25]. Other AEs of interest, including malignancy, MACE, opportunistic infections, and uveitis, were also uncommon through the end of the trials. Malignancy occurrence was comparable between placebo (0.4%) and golimumab (0.1%) during the placebo-controlled periods; no cases of lymphoma were reported through trial completion. While a small number of golimumab patients (*n* = 6 [0.4%]) acquired active TB in countries where TB is endemic (Argentina, Lithuania, Malaysia, Mexico, and Ukraine), no case of latent TB converted to active TB.

Among the three studies, AEs tended to be more common in patients with RA, with infections being the most common class of AE reported. Previous analyses of patients receiving TNFi have shown a higher incidence of serious infections in RA patients than in patients with AS or PsA [26, 27]. In the current study, patients in the RA study were, on average, older and had longer disease duration and were more likely to be receiving concomitant corticosteroids relative to patients in the PsA and AS studies. These factors have been previously associated with an increased risk of serious infection among RA patients treated with TNFi [28]. In addition, concomitant MTX use was required in the RA trial, but not in the other trials. In the PsA and AS trials, there was no clear pattern of differences in the rate of SAEs or serious infections between patients who were receiving concomitant MTX and those who were not. Elevations in ALT were more frequent in patients receiving concomitant MTX in the PsA trial, which was not unexpected considering that hepatic toxicity is commonly observed with MTX therapy. This pattern was not seen in the AS trial, although the proportion of patients receiving MTX in that trial was considerably lower.

Fewer than 3% of all golimumab-treated patients had an infusion reaction; none was serious or severe. Approximately 22% of patients treated with golimumab developed antibodies to golimumab, although only 6% of patients tested positive for neutralizing antibodies. The proportion of patients with antibodies to golimumab and neutralizing antibodies was numerically lower in patients who received concomitant MTX than in those who received golimumab monotherapy; however, interpretation of these results is limited by the lack of a golimumab monotherapy population in the RA study and the relatively small number of patients receiving golimumab+MTX in the AS study. Infusion reactions were more common in patients with antibodies to golimumab than those without antibodies to golimumab.

Oral corticosteroid use has been associated with an increased risk of serious infections in patients with RA, even with doses ≤ 5 mg/day (prednisone or equivalent) [29]. In the current pooled analyses, the proportions of patients with a serious infection during the placebo-controlled periods were numerically higher in patients receiving concomitant oral corticosteroids compared with patients not receiving these medications in both the placebo and golimumab groups. Among all golimumab-treated patients, the number of serious infections per 100 PY through study completion was similar for patients who did and did not receive oral corticosteroids. Previous research has shown that the highest risk of serious infection with TNFi tends to be within the first 6 months of treatment [28], and the risk of serious infections associated with corticosteroids is dose-dependent. The mean corticosteroid dose in the treatment groups across the three trials ranged from 6.1 to 7.9 mg/day (prednisone or equivalent), although patients could have received doses up to 10 mg/day. Also, it should be noted that there may have been imbalances in age, disease duration, and concomitant medication use between the patients who did and did not receive concomitant corticosteroids, as the studies were not designed to compare outcomes by corticosteroid use.

With data from over 700 golimumab-treated patients and approximately 71 patient-years of follow-up, these results represent the largest pooled analysis, to date, from phase 3 studies of IV golimumab in patients with RA, PsA, and AS. The studies included in the current analysis were limited to 1 or 2 years in duration, and the studies were not powered to detect rare events. Differences in study design also limited some of the analyses on concomitant medications. However, the totality of the pooled data across three rheumatologic indications demonstrated consistency with each of the individual trials.

## Conclusions

These pooled analyses from three double-blind, randomized, placebo-controlled, phase 3 trials of patients with RA, PsA, and AS included safety data from 1280 patients for a total of 1697 PY of follow-up. Infusion reactions were uncommon across the three studies. Concomitant use of MTX and of low-dose oral corticosteroids was associated with a higher occurrence of ALT elevations and serious infections, respectively. Overall, most elevations in liver transaminases were mild and transient, and few patients experienced a serious infection. The safety profile of IV golimumab was generally consistent across the RA, PsA, and AS trials, as well as with other TNFi.

## Data Availability

The data sharing policy of Janssen Pharmaceutical Companies of Johnson & Johnson is available at https://www.janssen.com/clinical-trials/transparency. As noted on this site, requests for access to the study data can be submitted through Yale Open Data Access (YODA) Project site at http://yoda.yale.edu.

## Abbreviations

AE: Adverse event
ALT: Alanine aminotransferase
AS: Ankylosing spondylitis
AST: Aspartate aminotransferase
BASDAI: Bath Ankylosing Spondylitis Disease Activity Index
BMI: Body mass index
CI: Confidence interval
CRP: C-reactive protein
EE: Early escape
GLM: Golimumab
IV: Intravenous
MACE: Major adverse cardiovascular events
MTX: Methotrexate
NSAIDs: Non-steroidal anti-inflammatory drugs
PBO: Placebo
PE: Primary endpoint
PsA: Psoriatic arthritis
PY: Patient years
R: Randomization
RA: Rheumatoid arthritis
SAE: Serious adverse event
SD: Standard deviation
TB: Tuberculosis
TNFα: Tumor necrosis factor alpha
TNFi: Tumor necrosis factor inhibitor
ULN: Upper limit of normal
